# Chemical Composition, *In Vitro* and *In Situ* Antimicrobial and Antibiofilm Activities of *Syzygium aromaticum* (Clove) Essential Oil

**DOI:** 10.3390/plants10102185

**Published:** 2021-10-15

**Authors:** Miroslava Kačániová, Lucia Galovičová, Petra Borotová, Veronika Valková, Hana Ďúranová, Przemysław Łukasz Kowalczewski, Hussein A. H. Said-Al Ahl, Wafaa M. Hikal, Milena Vukic, Tatsiana Savitskaya, Dzmitrij Grinshpan, Nenad L. Vukovic

**Affiliations:** 1 Institute of Horticulture, Faculty of Horticulture and Landscape Engineering, Slovak University of Agriculture, Tr. A. Hlinku 2, 94976 Nitra, Slovakia; l.galovicova95@gmail.com (L.G.); veronika.valkova@uniag.sk (V.V.); 2 Department of Bioenergy, Food Technology and Microbiology, Institute of Food Technology and Nutrition, University of Rzeszow, 4 Zelwerowicza Str., 35-601 Rzeszow, Poland; 3 AgroBioTech Research Centre, Slovak University of Agriculture, Tr. A. Hlinku 2, 94976 Nitra, Slovakia; petra.borotova@uniag.sk (P.B.); hana.duranova@uniag.sk (H.Ď.); 4 Department of Food Technology of Plant Origin, Poznań University of Life Sciences, 31 Wojska Polskiego Str., 60-624 Poznań, Poland; przemyslaw.kowalczewski@up.poznan.pl; 5 Medicinal and Aromatic Plants Research Department, National Research Centre, 33 El-Bohouth Str., Dokki, Giza 12622, Egypt; shussein272@yahoo.com; 6 Department of Biology, Faculty of Science, University of Tabuk, P.O. Box 741, Tabuk 71491, Saudi Arabia; wafaahikal@gmail.com; 7 Water Pollution Research Department, Environmental Research Division, National Research Centre, 33 El-Bohouth Str., Dokki, Giza, 12622, Egypt; 8 Department of Chemistry, Faculty of Science, University of Kragujevac, 34000 Kragujevac, Serbia; milena.vukic@pmf.kg.ac.rs; 9 Research Institute for Physical Chemical Problems, Belarusian State University, Leningradskaya Str. 14, 220030 Minsk, Belarus; savitskayaTA@bsu.by (T.S.); grinshpan@bsu.by (D.G.)

**Keywords:** *Syzygium aromaticum*, *in vitro*, *in situ*, antimicrobial activity, antibiofilm activity

## Abstract

The essential oil of *Syzygium* (*S.*) *aromaticum* (CEO) is known for its good biological activity. The aim of the research was to evaluate *in vitro* and *in situ* antimicrobial and antibiofilm activity of the essential oil produced in Slovakia. The main components of CEO were eugenol 82.4% and (E)-caryophyllene 14.0%. The antimicrobial activity was either weak or very strong with inhibition zones ranging from 4.67 to 15.78 mm in gram-positive and gram-negative bacteria and from 8.22 to 18.56 mm in yeasts and fungi. Among the tested bacteria and fungi, the lowest values of MIC were determined for *Staphylococcus* (*S.*) *aureus* and *Penicillium* (*P.*) *expansum*, respectively. The vapor phase of CEO inhibited the growth of the microscopic filamentous fungi of the genus *Penicillium* when tested *in situ* on bread. The strongest effect of mycelia inhibition in a bread model was observed against *P. expansum* at concentrations of 250 and 500 μL/mL. The best antimicrobial activity of CEO in the carrot model was found against *P. chrysosenum*. Differences between the mass spectra of *Bacillus* (*B.*) *subtilis* biofilms on the tested surfaces (wood, glass) and the control sample were noted from the seventh day of culture. There were some changes in mass spectra of *Stenotrophomonas* (*S.*) *maltophilia*, which were observed in both experimental groups from the fifth day of culture. These findings confirmed the impact of CEO on the protein structure of older biofilms. The findings indicate that, besides being safe and sensorially attractive, *S. aromaticum* has antimicrobial activity, which makes it a potential substitute for chemical food preservatives.

## 1. Introduction

*Syzygium aromaticum* is known for its use as spice in the preparation of food. Besides being valued for its flavoring properties, it can be used as an anti-cancer agent and a traditional remedy for many diseases such as asthma; digestive system, dental, and respiratory disorders; headaches; and sore throats [1]. It is also used in traditional medicine. Clove is widely used for treating dyspepsia, gastritis, and diarrhea. Furthermore, it was found that it has antipyretic, aphrodisiac, appetizing, expectorant, antiemetic, anxiolytic, myorelaxant, analgesic, decongestant, anti-inflammatory, and hypnotic effects [2]. The essential oil of *S. aromaticum* also shows anti-inflammatory, cytotoxic, and anesthetic activities besides the reported antimicrobial, antifungal, antiviral, antioxidant, and insecticidal properties [3].

The phenylpropene eugenol is the most important clove component. It is responsible for the characteristic strong aroma [4]. Inhibition of molds, yeast, and bacteria is one of the possible applications of clove essential oil [5]. The clove essential oil was proven to have repressing effects on some microorganisms such as *Alternaria* spp., *Aspergillus* spp., *Canninghamella* spp., *Lactobacillus* spp., *Fusarium* spp., *Clostridium* spp.; *Mucor* spp., *Salmonella* spp., *Penicillium* spp., and *Bacillus* spp. [6].

While being safe, non-toxic, and biodegradable, clove essential oil (CEO) shows a broad-spectrum antibacterial activity and can thus be used as bacteriostatic and anti-biofilm agent. Budri et al. [7] reported that CEO has significant inhibitory effect on biofilm. Rajkowska et al. [8] confirmed the inhibition of *Candida* biofilm on the surface of different materials by CEO. Adil et al. [9] proved that the main component of *C*EO may inhibit the adhesion of *S. mutans* to glass, prevent the formation of biofilm, and inhibit developed biofilm. While the formation of biofilm is a complex regulatory process, there are studies available that indicate the effectiveness of CEO as an antibiofilm agent.

The aim of the present study was to analyze the chemical composition and *in vitro* antimicrobial and antibiofilm activity of CEO. Molecular changes in bacterial biofilms were studied. The effectiveness of the gas phase of CEO against *Serratia* (*S.*) *marcescens* and *Penicillium* spp. was evaluated using food models.

## 2. Results

### 2.1. Chemical Composition of S. aromaticum EO

The essential oil of *S. aromaticum* was analyzed using gas chromatography with mass spectrometric (GC/MS) and flame ionization (GC-FID) detection. The main components were eugenol 82.4% and (E)-caryophyllene 14.0%. (Table 1). Based on the chemical composition, the analyzed oil was classified as thymol chemotype.

### 2.2. Antimicrobial Activity of S. aromaticum EO

The antimicrobial activity of CEO was evaluated by disk diffusion test and the results are presented in Table 2. A weak to moderate inhibitory activity of CEO was observed in the cases of most of the tested bacteria (including biofilm-forming bacteria), and yeast. *S. aureus* was the most susceptible of all the tested bacteria, with CEO showing a very strong inhibitory activity. MIC 50 and MIC 90 were determined by analysis of the minimum inhibitory concentrations. There were low values of MIC 50 (85.46 µL/mL), and MIC 90 (93.35 µL/mL) values were found for *S. aureus*. The highest MIC 50 and MIC 90 values were determined for *Y. enterocolitica*. Moderate MIC 50 and MIC 90 values were determined for *B. subtilis* and *B. subtilis* biofilm. Strong antimicrobial activity of CEO against all tested penicillia was observed, with *P. expansum* being the most susceptible with inhibition zone 18.56 mm and MIC 50 64.25 and MIC 90 75.12 µL/mL. Details of the results of antimicrobial activity and minimum inhibitory concentrations are given in Table 2.

### 2.3. Antimicrobial Analysis In Situ Using a Food Model

*In situ* antimicrobial analysis using bread showed that microscopic filamentous fungi of the genus *Penicillium* were inhibited by CEO vapors in all tested concentrations (Table 3). At the lowest concentration of the oil (62.5 µL/L), *P. glabrum* was the most strongly inhibited fungus (42.91%). The weakest inhibition at this concentration was observed for *P. commune.* The inhibition of *P. expansum* was 90.49% at the concentration of 125 μL/L and 100% at 250 μL/L and 500 µL/L. A significant difference in inhibition was observed for this species between 62.5 µL/L and the remaining concentrations. Significant differences in inhibition of *P. glabrum* and *P. chrysogenum* were found between 62.5 µL/L and 250 µL/L or 500 µL/L. These findings confirm the inhibitory effects of CEO against the potentially pathogenic fungi tested.

The effect of the vapor phase of CEO against *P. chrysogenum*, *P. expansum* was recorded in *in situ* analysis using carrots at all tested concentrations, while *S. marcescens* was only inhibited when the highest concentration of the oil was used (Table 4). The highest inhibition of *P. chrysogenum* (93.53%) and *P. expansum* (75.94%) was observed at the concentration of 500 µL/L. The lowest concentration of CEO yielded the lowest inhibition results for both molds. The vapor phase of CEO was shown to have the best inhibitory effect on penicillia in the *in situ* tests with the food model.

### 2.4. Analysis of Biofilm Developmental Phases and Evaluation of Molecular Differences on Different Surfaces Using MALDI-TOF MS Biotyper

**Figure 1:**
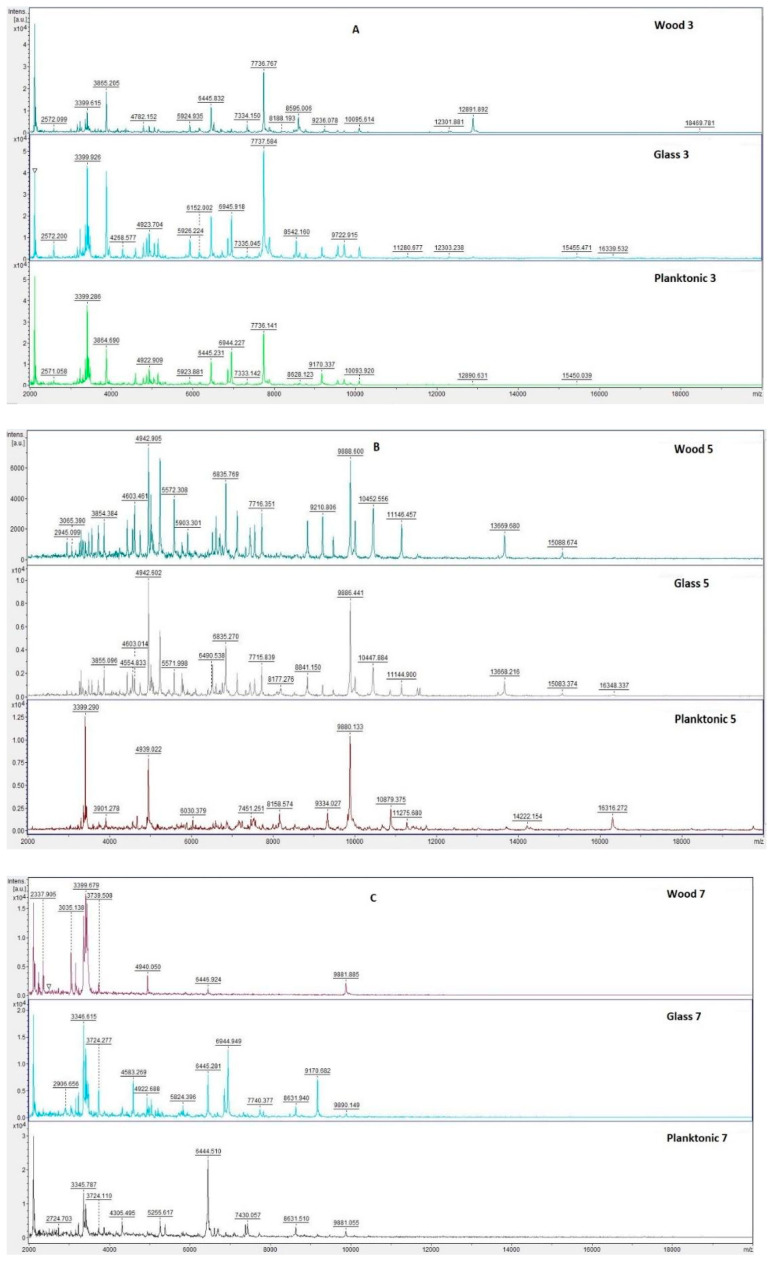

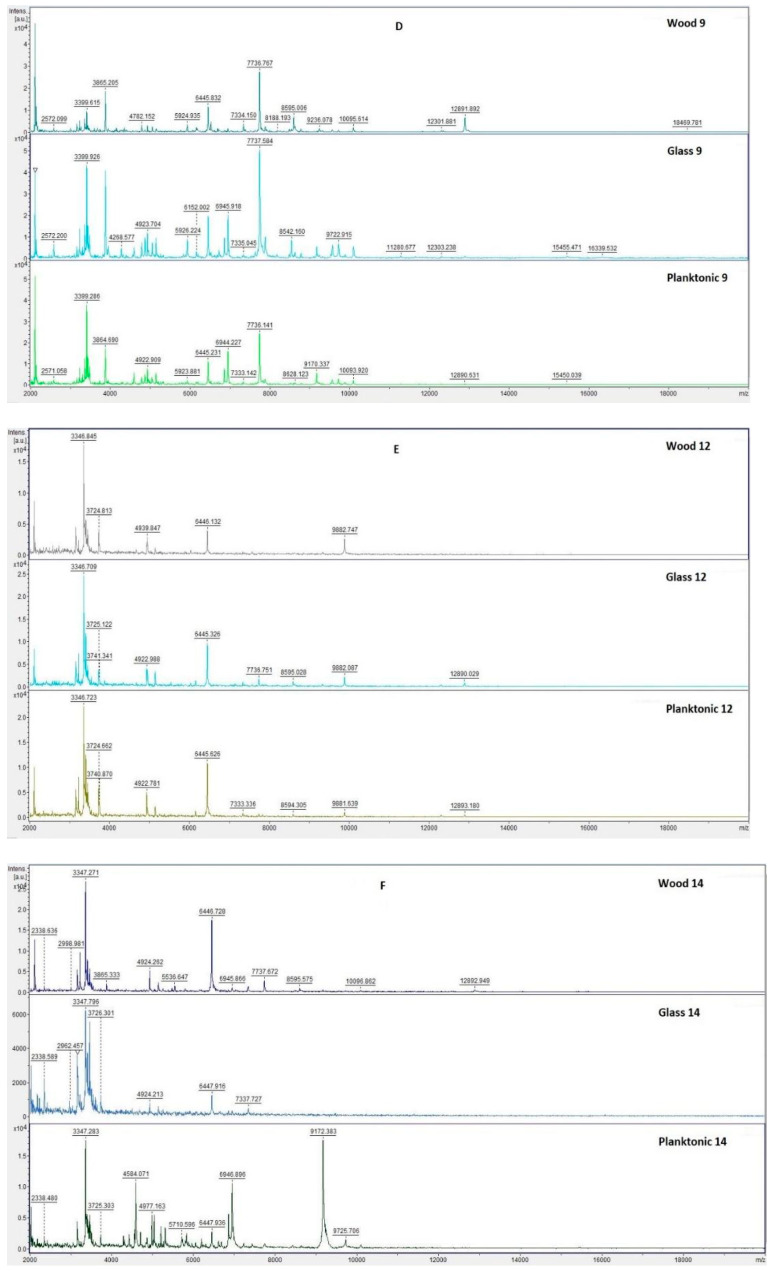
MALDI-TOF mass spectra of *B. subtilis* biofilm during development after the addition of CEO: (**A**) 3rd day, (**B**) 5th day, (**C**) 7th day, (**D**) 9th day, (**E**) 12th day, and (**F**) 14th day.

The effect of CEO on *B. subtilis* and *S. maltophilia* biofilms was analyzed using MALDI TOF MS Biotyper in order to observe changes in molecular structure that accompany growth inhibition. The spectra of biofilms and planktonic cells in the control group developed identically and, therefore, the spectra of planktonic cells were used instead of the control spectrum for improved clarity. The two experimental spectra from different surfaces (glass, wood) and the planktonic spectrum representing the development of the control group are shown for each day of the experiment. Figure 1 shows the mass spectra of *B. subtilis* biofilm during individual days of the experimental evaluation.

Mass spectra after days 3 and 5 (Figure 1A,B) of the culture showed the same peaks, indicating production of the same proteins by new biofilms and control planktonic cells. There were no changes observed in bacterial cultures at the protein level. Differences between the mass spectra of biofilms on the tested surfaces (wood, glass) and the control sample stared to emerge on day 7 (Figure 1C–F). This indicated changes in the protein profile of the CEO-treated biofilm. It seems that CEO can influence the homeostasis of a bacterial biofilm formed on wooden and glass surfaces.

**Figure 2:**
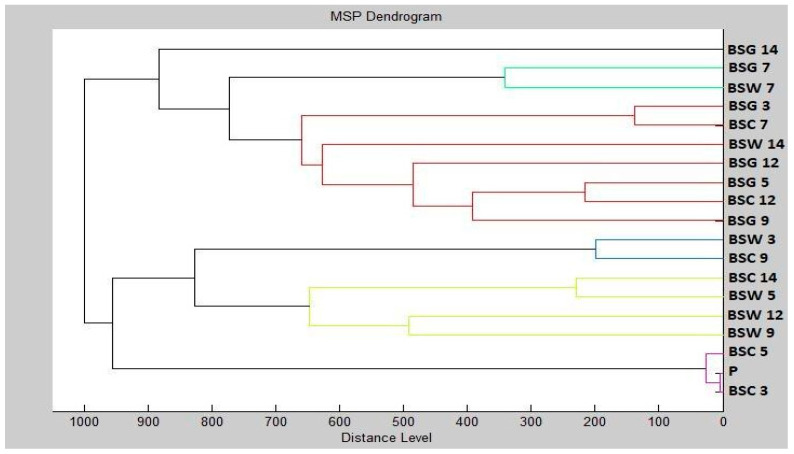
Dendrogram of *B. subtilis* generated using MSPs of the planktonic cells and the control. BS, *B. subtilis*; C, control; G, glass; W, wood; and P, planktonic cells.

Using the data obtained with mass spectrometry, a dendrogram was created to visualize similarities in biofilm structure that are based on MSP distance (Figure 2). It can be stated from the dendrogram that the planktonic stage (P) together with the control groups and new biofilms had the shortest distance during the third and fifth days (BSG 3, BSW 3, BSG 5, BSW 5). The similarity in the protein profile of the control groups was confirmed by the short distances of the MSP. New biofilms and control planktonic cells also showed short MSP lengths that corresponded to similar mass spectra. The distance of the experimental groups of SMEs increased gradually over time. The mass spectra analyzed on the 12th and 14th days of the experiment had the longest MSP lengths, indicating changes in the molecular profile of *B. subtilis*.

**Figure 3:**
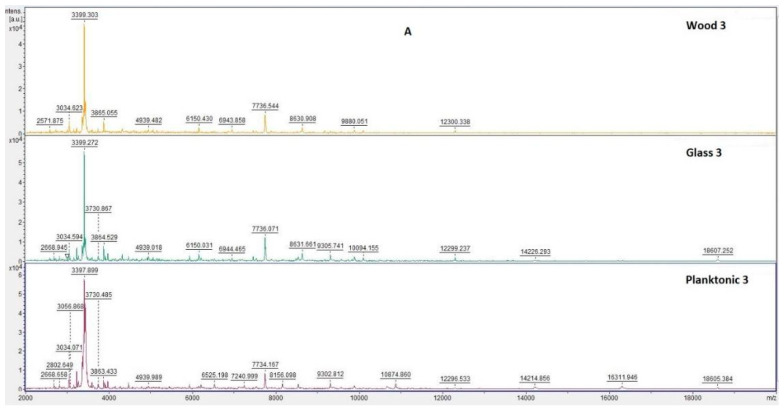

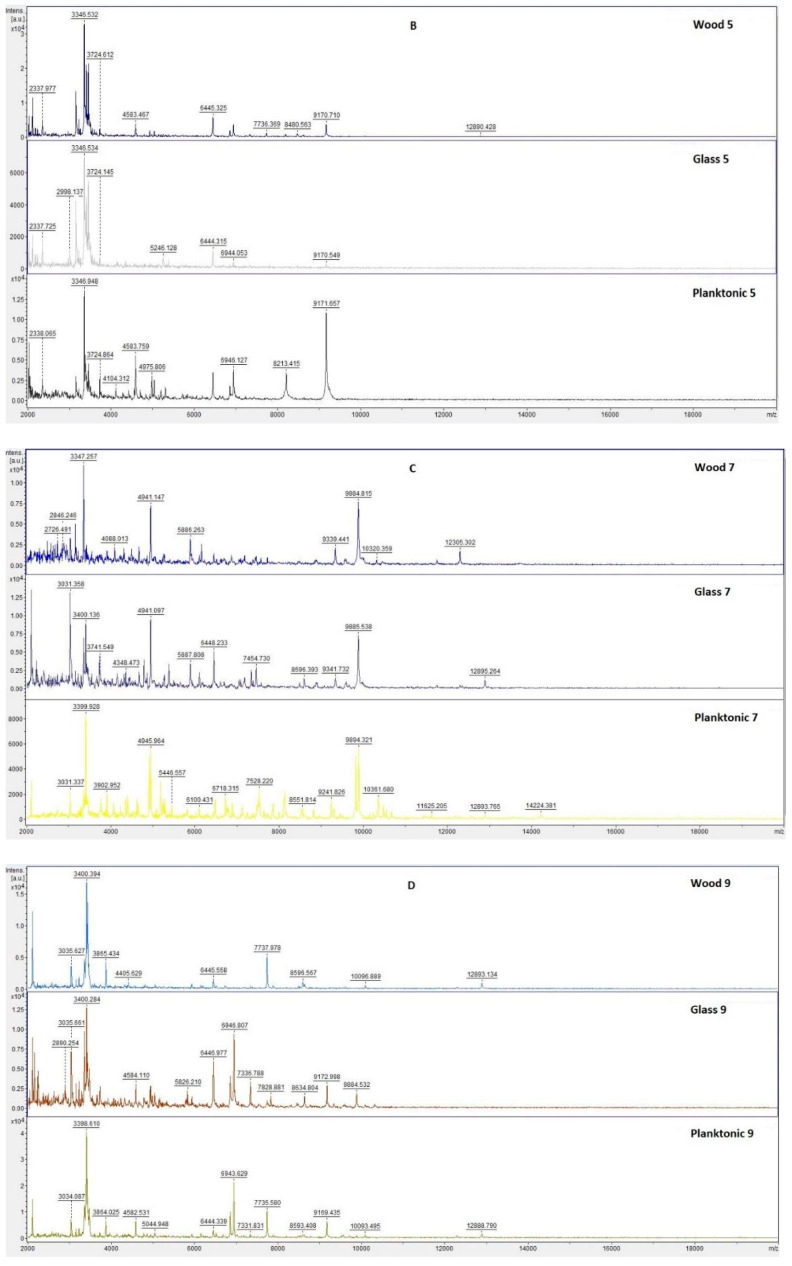

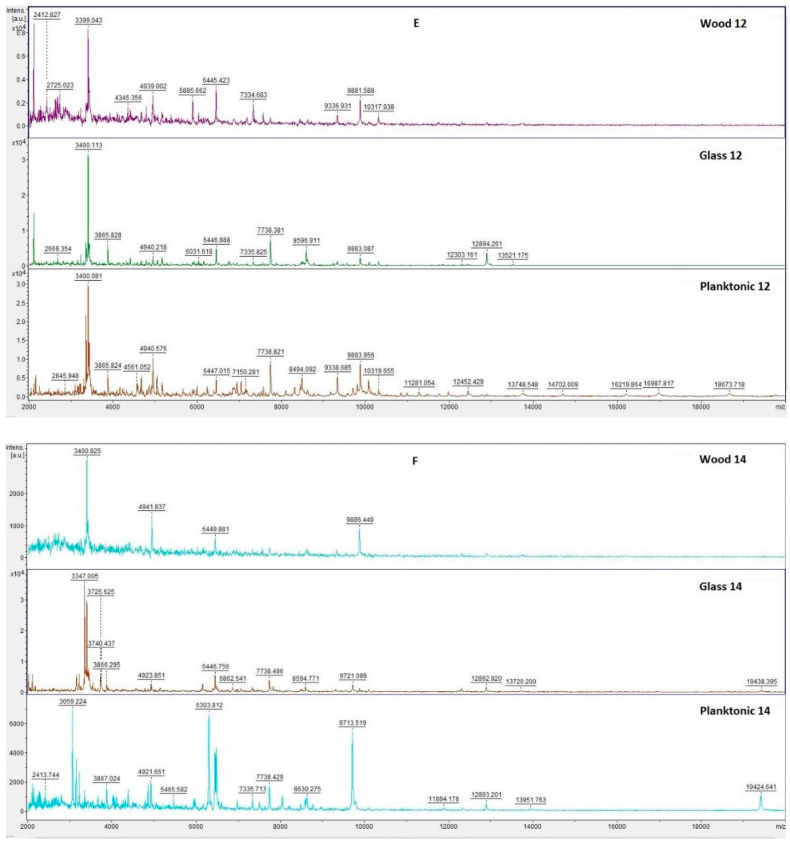
MALDI-TOF mass spectra of *S. maltophilia* during biofilm development: (**A**) 3rd day, (**B**) 5th day, (**C**) 7th day, (**D**) 9th day, (**E**) 12th day, and (**F**) 14th day.

Figure 3 shows the spectra of developmental stages of *S. maltophilia* biofilm over the entire duration of the experiment.

Analysis of *S. maltophilia* protein spectra by MALDI-TOF MS Biotyper showed similarity between the experimental spectra and the control planktonic spectrum on the third day of the experiment (Figure 3A), indicating that the bacterial cells in biofilm developed similarly to the planktonic cells. Changes in mass spectra were observed in both experimental groups from the fifth day (Figure 3B–F). This finding confirms the effect of CEO on the protein structure of older biofilms.

**Figure 4:**
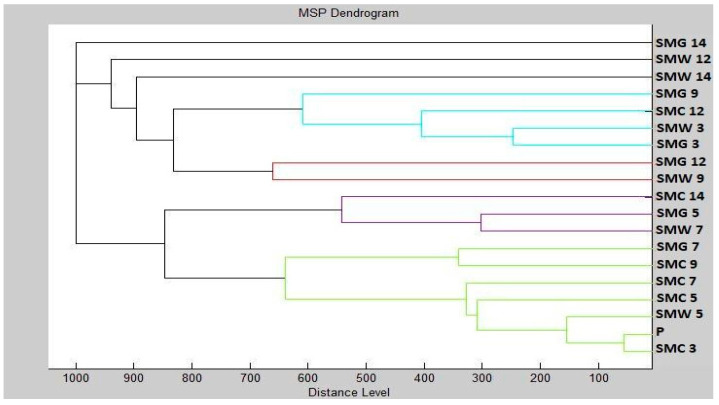
Dendrogram of *S. maltophilia* generated using MSPs of the planktonic cells and the control. SM, *S. maltophilia*; C, control; G, glass; W, wood; and P, planktonic cells.

Mass spectra dendrogram also confirmed the similarity of the new biofilms to planktonic cells and control cells (Figure 4). The changing distance of MSP during the progression of the experiment indicates differences in protein production due to the effect of the addition of CEO.

## 3. Discussion

Essential oils are potential sources of novel antimicrobial compounds, especially those active against bacterial pathogens and microscopic filamentous fungi. Chemical composition analysis revealed that the tested essential oil was of the eugenol chemotype. Eugenol was the major compound (90.3%), accompanied by *β*-caryophyllene (4.83%) and eugenol acetate (1.87%), in an essential oil extracted from the south of Brazil [10]. In another study where essential oil of clove from the south of Brazil was analyzed, greater quantities of caryophyllene (39.63%) and less of eugenol (56.06%) were detected [11]. CEO from China [12] and Italy [13] also contained eugenol as their major compound, with 90.84% and 77.9%, respectively. Similar amounts were found in the present study. The amounts of eugenol and accompanying compounds in the essential oil can be directly related to the different geographic areas of origin of the plant material. They can also be influenced by biotic and abiotic factors such as seasonality, stage of development, age of the plant, and climatic conditions [14]. In addition, the extraction method, such as distillation, which is used for obtaining the oil, can also have an effect on its chemical composition. Moreover, it was found that storage conditions may influence the content of its volatile components [15].

CEO, which is used as an antiseptic in oral infections, inhibits Gram-negative and Gram-positive bacteria as well as microscopic filamentous fungi. There are many reports that prove that clove oil and its main active component, eugenol, affect common, food-derived, Gram-negative bacteria such as *Escherichia coli*, *Salmonella*, *P. aeruginosa*, etc., and Gram-positive bacteria such as *Staphylococcus*, *Streptococcus*, *Listeria*, etc. This conclusion is based on the reported inhibiting effects on migration, adhesion, expression of virulence factors, and biofilm formation in these bacteria. Clove oil and eugenol have good prospects for application in the food antisepsis field [16]. With the disk diffusion method, we found the antimicrobial activity against the tested microorganisms, including biofilm-forming bacteria, to be either weak or very strong. *S. aureus* was found to be the most susceptible to CEO among the bacteria tested. In another study, CEO was active against standard strains of Gram-negative and Gram-positive bacteria, showing inhibition zones between 14 and 25 mm in the disk diffusion test [17]. The authors reported intermediate bacterial activity of the oil against food bacteria with inhibition diameters of 14.6 ± 1.7 mm for *P. putida*, 15 ± 0.66 mm for *E. coli*, and 15.6 ± 0.4 mm for *S. aureus*. In a different research, inhibition zones ranging from 21.3 ± 2.2 mm for *S. aureus* ATCC and 35.6 ± 2.7 mm for *P. aeruginosa* ATCC were observed [18]. The results of our study differed from the ones referenced where the zones of inhibition were generally smaller. Cimanga et al. [19] reported that CEO could be a strong inhibitor of *S. aureus* and *E. coli* and a moderate inhibitor of *P. aeruginosa*. Using the microdilution method, Gislene et al. [20], Hili et al. [21], and Nzeako et al. [22] found that CEO manifested antimicrobial activity against *S. aureus*, *E. coli*, and *C. albicans* at various concentrations of the extracts. The sensitivity to the antimicrobial effect of CEO (in descending order) was *E. coli* with MIC 2 µL/mL, *C. albicans* with MIC 16 µL/mL, *E. faecalis* with MIC 32 µL/mL, and *S. aureus* with MIC 32 µL/mL [23]. Different results were found using microdilution methods in our study. Clove oil has a strong antimicrobial activity. Its minimum inhibitory concentration in the range of 0.2–0.625 mg/mL was recorded against the bacteria *S. aureus*, *Staphylococcus epidermidis*, *E. coli*, and *P. aeruginosa* [24,25,26].

From among the microscopic fungi tested in our study, *P. expansum* was found to be the most susceptible to CEO. Among the tested yeasts, *C. krusei* was the least resistant. Eugenol from the clove is the essential ingredient that is responsible for its antifungal activity [27]. A strong antifungal activity of CEO was reported against opportunistic fungal pathogens such as *C. albicans*, *Cryptococcus neoformans*, and *Aspergillus fumigatus*. A considerable variation in inhibition zone sizes, ranging from 12 to 22 mm, was observed among fungal isolates depending on their sensitivity to CEO [28]. Nunez et al. [29] reported a strong fungicidal effect of eugenol against *C. albicans*, *P. citrinum*, *A. niger*, and *T. mentagrophytes*. Ahmad et al. [30] also reported strong antifungal activity of clove oil against *C. albicans*, *C. neoformans*, and *A. fumigatus*. Pinto et al. [31] determined MIC to evaluate the antifungal activity of the clove oil and its main component, eugenol, against *Candida*, *Aspergillus*, and clinical and ATCC strains of dermatophytes. Similar to our results, the essential oil and eugenol showed inhibitory activity against all the tested strains. Chami et al. [32] confirmed carvacrol and eugenol as strong potential antifungal agents and suggested their use as therapeutic agents for oral candidiasis.

The effect of the vapor phase of CEO against *P. chrysogenum* and *P. expansum* was recorded at all tested concentrations in *in situ* analysis using carrots. Noteworthy, *S. marcescens* was inhibited only when the oil was applied in the concentration of 500 µL/L. The investigation of inhibition of the microscopic filamentous by vapor phase CEO also indicated the susceptibility of penicillia. In the bread model studies, the best inhibition result was observed against *P. expansum* at concentrations of 250 and 500 µL/mL. In the carrot model studies, *P. chrysogenum* was found to be the most susceptible. There exists a different study in which the antifungal activities of CEO and its volatile vapor against dermatophytic fungi, including *C. albicans*, *Epidermophyton floccosum*. *Microsporum audouinii*, *Trichophyton mentagrophytes*, and *Trichophyton rubrum*, were studied. Spore germination and mycelial growth were found to be strongly inhibited by both the oil and its volatile vapor. Moreover, the volatile vapor of CEO showed fungistatic activity, whereas the direct application of the oil resulted in a fungicidal effect [33]. Jain and Agrawal [34] noticed fungistatic activity of volatile vapor of several essential oils. No other authors focused on antifungal activity of volatile vapor of clove oil. There are studies in which the differences in inhibitory activity of essential oils in different food model systems were investigated [35]. It was found that CEO did not have a significant effect on the count of *E. coli* O157: H7 relative to control; however, it showed significant inhibitory effect in blanched spinach at similar concentration [36]. Aguilar-Gonzalez et al. [37] evaluated the antifungal activity of the vapor phase of clove and mustard EOs, individually and in combination, against gray mold (*Botrytis cinerea*) in strawberries, and noted inhibitory activity.

Bacterial biofilms can be characterized as non-homogenous structures with a high surface roughness, diversification of cells’ metabolic activity, and presence of extracellular polymeric substances as well as cells’ and spores’ liberation from the upper parts of the biofilm matrix [38]. The difference between the mass spectra of *B. subtilis* biofilms on the tested surfaces (wood, glass) and the control sample was noticeable from the seventh day in our study. There were some changes in mass spectra of *S. maltophilia* in both experimental groups from the fifth day. These results confirmed the effect of CEO on the protein structure of older biofilms. CEO is one of the most bioactive essential oils due to high antimicrobial activity of eugenol [39]. The main compound of the oil used in the presented research was eugenol, which constituted 86.99% of it. As demonstrated in another study, the activity of CEO against *Alicyclobacillus* biofilm may be related to both its bactericidal effect, resulting in a decrease in the number of planktonic cells, and the changes in the adherence capability of cells [40]. MALDI-TOF MS Biotyper has only been used in a few cases. Li et al. [41] analyzed *B. subtilis* biofilm and determined the spatial distribution of specific peptides and lipopeptides that are produced in biofilms by using this method. Kubesová et al. [42] used MALDI-TOF MS to analyze the biofilm produced by the genus *Candida*. Rams et al. [43] found out that the phenotypic identification of culturable *P. gingivalis* biofilms is 100% accurate using MALDI-TOF MS because of the differences in the protein profile. The changes in the mass spectra profile were demonstrated by MALDI-TOF in Kačániová et al. [44,45], where inhibitory effects on the biofilm of *Coriandrum sativum* and *Citrus aurantium* EOs were detected. The use of MALDI-TOF can be a fast and easy method for the assessment of the biofilm growth and degradation due to structural and molecular changes.

## 4. Materials and Methods

### 4.1. Essential Oil

The essential oil of *S. aromaticum* was obtained from Hanus, s.r.o. (Nitra, Slovakia). It was a product of steam distillation of fresh leaves from 2021. It was stored in the dark at 4 °C for the whole time. The analyses were carried out in 2021.

### 4.2. Chemical Characterization of Essential Oil Samples by Gas Chromatography/Mass Spectrometry (GC/MS) and Gas Chromatography (GC-FID)

GC/MS analysis of CEO was performed using an Agilent 6890N gas chromatograph (Agilent Technologies, Santa Clara, CA, USA) coupled to a quadrupole mass spectrometer 5975B (Agilent Technologies, Santa Clara, CA, USA) equipped with an HP-5MS capillary column (30 m × 0.25 mm × 0.25 µm). The temperature ramp was from 60 °C to 150 °C (increasing rate 3 °C/min) and from 150 °C to 280 °C (increasing rate 5 °C/min). The total run time of the program was 60 min. Helium 5.0 was used as the carrier gas with a flow rate of 1 mL/min. The injection volume was 1 µL (EO sample was diluted in pentane), while the split/splitless injector temperature was set at 280 °C. Split mode injection with split ratio at 40.8:1 was used. The electron-impact mass spectrometric data (EI-MS; 70 eV) were acquired in scan mode over the m/z range 35–550. MS ion source and MS quadrupole temperatures were 230 °C and 150 °C, respectively. Acquisition of data started after 3 min of solvent delay time.

GC-FID analyses were performed on Agilent 6890N gas chromatograph coupled to FID detector. Column (HP-5MS) and chromatographic conditions were the same as for GC-MS. The temperature of the FID detector was set at 300 °C.

The individual volatile constituents of *S. aromaticum* EO sample were identified according to their retention indices [46] and they were compared with the reference spectra (Wiley and NIST databases). The retention indices were determined experimentally by standard method, which included retention times of n-alkanes (C6–C34), injected under the same chromatographic conditions [47]. The percentages of the identified compounds (amounts higher than 0.1%) were derived from their GC peak areas.

### 4.3. Tested Microorganisms

Gram-positive bacteria (*B. subtilis* CCM 1999, *E. faecalis* CCM 4224, *S. aureus* subsp. *aureus* CCM 8223), gram-negative bacteria (*P. aeruginosa* CCM 3955, *S. enterica* subsp. *enterica* ser. Enteritidis CCM 4420, *S. marcescens* CCM 8588 *Y. enterocolitica* CCM 7204), and yeasts (*C. krusei* CCM 8271, *C. albicans* CCM 8261, *C. tropicalis* CCM 8223, *C. glabrata* CCM 8270) were obtained from the Czech collection of microorganisms (Brno, Czech Republic). The biofilm-forming bacteria *B. subtilis* and *S. maltophilia* were obtained from the dairy industry. Bacteria were identified with 16S rRNA sequencing and MALDI-TOF MS Biotyper. The fungi *P. glabrum*, *P. chrysogenum*, *P. expansum*, and *P. commune* were obtained from grape samples, and were identified by 16S rRNA sequencing and MALDI-TOF MS Biotyper.

### 4.4. Antimicrobial Activity: Disc Diffusion Method

Antimicrobial activity of *S. aromaticum* EO was determined by the disc diffusion method. The microbial inoculum was cultivated for 24 h on Tryptone soya agar (TSA, Oxoid, Basingstoke, UK) at 37 °C for bacteria and Sabouraud Dextrose Agar (SDA, Oxoid, Basingstoke, UK) at 25 °C for yeasts. The inoculum was adjusted to optical density 0.5 McFarland standard (1.5 × 10^8^ CFU/mL) and 100 μL was added on plates with Mueller Hinton agar (MHA, Oxoid, Basingstoke, UK). Sterile 6-mm discs saturated with 10 μL of *S. aromaticum* EO were placed on the layer of agar containing the suspension of microorganisms. The samples were incubated for 24 h at 37 °C for bacteria and 25 °C for yeasts. Two antibiotics (Cefoxitin, Gentamicin, Oxoid, Basingstoke, UK), and one antifungal (Fluconazole, Oxoid, Basingstoke, UK) were used as positive controls for gram-negative and gram-positive bacteria and yeasts, respectively. Disks impregnated with 0.1% DMSO (dimethylsulfoxid, Centralchem, Bratislava, Slovakia) served as negative control. The antimicrobial activity was categorized as either very strong, moderate, or weak when the zone of growth inhibition was larger than 15, 10, and 5 mm, respectively. The analyses were performed in triplicate.

### 4.5. Minimum Inhibitory Concentrations (MIC)

Microbial inoculum was cultivated for 24 h in Mueller Hinton Broth (MHB, Oxoid, Basingstoke, UK) at 37 °C for bacteria and Sabouraud Dextrose Broth (SDB, Oxoid, Basingstoke, UK) at 25 °C for yeasts. An aliquot of 50 μL of inoculum with an optical density of 0.5 McFarland was added to a 96-well microtiter plate. Subsequently, the *S. aromaticum* EO was prepared by serial dilution to a concentration range from 400 μL/mL to 0.2 μL/mL in MHB/SDB, and 100 μL of suspension was thoroughly mixed with bacterial inoculum in wells. Bacterial samples were incubated for 24 h at 37 °C. Yeast samples were incubated for 24 h at 25 °C. MHB/SDB with EO was used as a negative control and MHB/SDB with inoculum was used as positive control, representing uninhibited growth.

For non-adherent microorganisms, the absorbance was measured after an incubation period at 570 nm by Glomax spectrophotometer (Promega Inc., Madison, WI, USA). The MIC of biofilm-forming bacteria was measured with the use of crystal violet. The suspension with non-attached cells was discarded, the wells were washed with distilled water three times, and left to dry at room temperature. Following the addition of 200 μL of 0.1% (*w*/*v*) crystal violet to the wells, the plates were incubated for 15 min. Subsequently, the wells were repeatedly washed and dried. Stained biofilms were solubilized with 200 μL of 33% acetic acid [48], and absorbance was measured at 570 nm. Minimum inhibitory concentration was determined as the concentration of CEO at which absorbance was lower than the absorbance of the maximal growth control. The test was performed in triplicate.

### 4.6. Antimicrobial Analysis In Situ on a Food Model

Antifungal effect of *S. aromaticum* EO vapor phase was evaluated in 0.5-L sterile, glass jars (Bormioli Rocco, Fidenza, Italy) on a bread surface used as a food model. The fungi of *Penicillium* genus were cultivated for 5 days on Sabouraud Dextrose agar (SDA, Oxoid, Basingstoke, UK) at the temperature 25 °C. The cultures were applied to the bread slices (15 × 15 × 1.5 cm) by three stabs. A 6-cm sterile, filter paper was placed to the jar lid and 100 µL of CEO (62.5, 125, 250, and 500 µL/L diluted in ethyl acetate) were applied. The control group was left untreated. The jars were hermetically sealed and they were incubated in the dark for 14 days at 25 °C ± 1 °C.

Antibacterial analysis of the vapor phase of CEO *in situ* was tested on *S. marcescens*. Warm MHA was poured into 60-mm Petri dishes (PD) and in the lid too. Sliced carrots (0.5 mm) were placed on agar. Then, an inoculum was prepared, as previously described. CEO was diluted in ethyl acetate to 500, 250, 125, and 62.5 μL/L and applied to sterile filter paper. After evaporation of the remaining ethyl acetate (1 min), the dishes were sealed and incubated at 37 °C for 7 days.

Inhibition of the fungal growth was evaluated by stereological methods. Volume density (Vv) of fungi was estimated using ImageJ software. The stereological grid points of the colonies (P) and substrate (p) were counted. The density of fungal growth was calculated as percentage (%) according to the formula Vv = P/p × 100. The antifungal activity of EO was expressed as mycelial growth inhibition in percentage (%) (MGI): MGI = [(C − T)/C] × 100, where C was density of the fungal growth in the control group and T was density of the fungal growth in the treatment group [49,50].

*In situ* bacterial growth was determined using stereological methods. In this concept, the volume density (Vv) of bacterial colonies was first estimated using ImageJ software counting the points of the stereological grid hitting the colonies (P) and those (p) falling to the reference space (growth substrate used). The volume density of bacterial colonies was consequently calculated as follows: Vv (%) = P/p. The antibacterial activity of EO was defined as the percentage of bacterial growth inhibition (BGI) BGI = [(C − T)/C] × 100, where C and T were bacterial growth (expressed as Vv) in the control group and the treatment group, respectively. Negative results represented growth stimulation.

### 4.7. Analysis of Differences in Biofilm Development with MALDI-TOF MS Biotyper

The changes of protein spectra during biofilm development after CEO addition were evaluated by MALDI-TOF MS Biotyper. *S. maltophilia* was used as a representative of gram-negative biofilm-forming bacteria and *B. subtilis* was representative of gram-positive biofilm-forming bacteria, and both bacterial strains were isolated from the milk industry. The biofilm-forming bacteria were added to 50-mL polypropylene tubes with 20 mL of MHB; subsequently, a wooden toothpick and a glass slide were added as models of different surfaces. The experimental groups were treated with 0.1% CEO, and control group samples were left untreated. The samples were incubated at 37 °C on a shaker at 170 rpm. The samples were analyzed after 3, 5, 7, 9, 12, and 14 days. The biofilm samples were taken from a glass slide and wooden toothpick with a sterile cotton swab, and they were imprinted onto a MALDI TOF metal target plate. The planktonic cells were obtained from 300 µL of culture medium, cells were centrifuged for 1 min at 12,000 rpm, and the supernatant was discarded. The pellet was resuspended in 30 μL of ultrapure water and the suspension was centrifuged for 1 min at 12,000 rpm. This cycle was repeated three times. Then 1 μL of the so-prepared planktonic cell suspension was applied to a target plate. The target plate was dried and 1 μL of α-Cyano-4-hydroxycinnamic acid matrix (10 mg/mL) was applied. The samples were processed with MALDI-TOF MicroFlex (Bruker Daltonics) linear and positive mode for the range of m/z 200–2000 after crystallization. The spectra were obtained by an automatic analysis and the same sample similarities were used to generate the standard global spectrum (MSP) and the 19 MSP was generated from the spectra by MALDI Biotyper 3.0 and were grouped into dendrograms using Euclidean distance [44].

### 4.8. Statistical Data Evaluation

SAS^®^ software version 8 was used for data processing. The results of the MIC value (concentration that caused 50% and 90% inhibition in bacterial growth) were determined by logit analysis.

## 5. Conclusions

The main components of the essential oil of *S. aromaticum* were eugenol 82.4% and (E)-caryophyllene 14.0%. A very strong antifungal effect of the vapor phase of the essential oil against the genus *P. expansum* was observed in the experiments that employed the food model (bread). Strong inhibiting effects of the vapor phase against *P. chrysogenum* were also noted in the test with carrots. The results indicate that CEO has potential for future use in extension of the shelf life of bakery products and that it could find application in the storage of root vegetables. The tested oil had moderate antimicrobial as well as antibiofilm effects that were observed on various surfaces and detected by MALDI-TOF MS Biotyper. In the case of EOs with a dominant proportion of volatile components, a stronger effect was often observed in the vapor phase in comparison to direct contact application. The decrease in microbial population caused by the addition of CEO depended on the concentration, and the inhibitory activity of the oil was lower in food systems when compared to *in vitro* systems.

## Figures and Tables

**Table 1:** Chemical composition of essential oil from *S. aromaticum.*

No	RI ^a^	Compound ^b^	% ^c^
1	830	furfural	Tr
2	983	6-methyl-5-hepten-2-one	Tr
3	1190	methyl salicylate	Tr
4	1193	2-allyl-phenol	Tr
5	1360	eugenol	82.4
6	1379	a-copaene	Tr
7	1422	(E)-caryophyllene	14.0
8	1456	a-humulene	1.8
9	1519	eugenol acetate	0.9
10	1583	caryophyllene oxide	0.7
11	1755	benzyl benzoate	Tr
	total		99.7

^a^ Values of retention indices on HP-5MS column; ^b^ identified compounds; ^c^ tr—compounds identified in amounts less than 0.1%.

**Table 2:** Antimicrobial activity of *S. aromaticum* essential oil.

Microorganism	Zone Inhibition (mm)	Activity of EO	MIC 50 (µL/mL)	MIC 90 (µL/mL)	ATB
*S. enteritidis*	9.44 ± 1.01	*	216.23	265.41	29.00 ± 0.10
*P. aeruginosa*	5.22 ± 1.20	*	223.38	284.56	28.00 ± 0.06
*Y. enterocolitica*	7.67 ± 1.41	*	224.46	278.92	28.00 ± 0.05
*S. marcescens*	10.44 ± 1.01	**	112.13	145.25	27.00 ± 0.02
*S. aureus*	15.78 ± 0.67	***	85.46	93.35	29.00 ± 0.03
*B. subtilis*	11.22 ± 0.83	**	121.13	138.91	26.00 ± 0.05
*E. faecalis*	4.67 ± 1.32	*	218.36	264.55	29.00 ± 0.03
*C. albicans*	8.22 ± 1.53	*	221.43	226.32	28.00 ± 0.06
*C. krusei*	9.89 ± 1.76	*	211.82	265.33	26.00 ± 0.12
*C. tropicalis*	9.33 ± 0.58	*	214.54	271.36	29.00 ± 0.02
*C. glabrata*	9.33 ± 1.00	*	217.22	264.36	26.00 ± 0.03
Biofilm *S. maltophilia*	8.89 ± 1.36	*	223.18	284.32	27.00 ± 0.05
Biofilm *B. subtilis*	10.44 ± 0.88	**	103.64	128.64	26.00 ± 0.03
*P. commune*	16.52 ± 0.15	***	74.32	81.26	25.00 ± 0.05
*P. expansum*	18.56 ± 0.22	***	64.25	75.12	26.00 ±0.11
*P. glabrum*	18.32 ± 0.21	***	68.41	78.12	27.00 ± 0.06
*P. chrysogenum*	17.36 ± 0.08	***	75.11	86.92	26.00 ± 0.09

* Weak antimicrobial activity (zone 5–10 mm). ** Moderate inhibitory activity (zone > 10 mm). *** Very strong inhibitory activity (zone > 15 mm). ATB—antibiotics, positive control (Cefoxitin for G^−^, Gentamicin for G^+^, Fluconazole for yeast and fungi).

**Table 3:** *In situ* analysis of the antifungal activity of the vapor phase of CEO in bread.

Mycelial Growth Inhibition [%]				
Fungi	Concentration of EO			
	62.5 µL/L	125 µL/L	250 µL/L	500 µL/L
*P. commune*	16.41 ± 6.84 ^a^	45.72 ± 5.49 ^b^	96.22 ± 1.20 ^c^	98.78 ± 1.72 ^cd^
*P. expansum*	16.73 ± 6.38 ^a^	90.49 ± 12.26 ^b^	100.00 ± 0.00 ^bc^	100.00 ± 0.00 ^bd^
*P. glabrum*	42.91 ± 5.87 ^a^	54.50 ± 9.01 ^a^	93.42 ± 9.30 ^b^	100.00 ± 0.00 ^bc^
*P. chrysogenum*	30.31 ± 9.70 ^a^	12.21 ± 7.87 ^a^	97.90 ± 1.49 ^b^	99.45 ± 0.78 ^bc^

Means ± standard deviation. Values followed by different superscript within the same row are significantly different (*p* < 0.05).

**Table 4:** Results of *in situ* analysis of antimicrobial activity of the vapor phase of CEO on carrots.

Bacterial Growth Inhibition [%]				
Microorganisms	Concentration of EO			
	62.5 µL/L	125 µL/L	250 µL/L	500 µL/L
*P. chrysogenum*	16.93 ± 0.37 ^a^	51.04 ± 4.43 ^ab^	61.22 ± 15.73 ^bc^	93.53 ± 5.11 ^c^
*P. expansum*	19.43 ± 0.89 ^a^	59.81 ± 4.33 ^b^	64.98 ± 2.81 ^b^	75.94 ± 0.91 ^c^
*S. marcescens*	0.00 ± 0.00 ^a^	0.00 ± 0.00 ^a^	0.00 ± 0.00 ^a^	49.11 ± 13.73 ^b^

Means ± standard deviation. MGI: mycelial growth inhibition, BGI: bacterial growth inhibition; values followed by different superscript within the same row are significantly different (*p* < 0.05).

## Data Availability

Data are contained within the article.
